# Vancomycin vs metronidazole use for the treatment of *Clostridioides difficile* infection in a tertiary care hospital in Saudi Arabia

**DOI:** 10.1016/j.heliyon.2023.e22053

**Published:** 2023-11-04

**Authors:** Abrar F. Alhameed, Nada Saferuddin, Tariq Alturkistani, Mohammed Al Musawa, Nader Damfu, Majda Alattas

**Affiliations:** a Pharmaceutical Care Division, King Faisal Specialist Hospital and Research Centre, Madinah, Saudi Arabia; b Pharmaceutical Care Division, King Faisal Specialist Hospital and Research Centre, Jeddah, Saudi Arabia; c Pharmaceutical Care Department, King Abdul Aziz Medical City, Jeddah, Saudi Arabia

## Abstract

**Background:**

The 2017 Infectious Diseases Society of America (IDSA) *Clostridioides difficile* infection (CDI) guidelines recommendation for oral vancomycin as preferred treatment was based on studies conducted in North America, Australia, and Europe. According to recent published data, metronidazole remains a reasonable option. No studies have been conducted in Saudi Arabia to compare prescribing patterns before and after the release of the guidelines. Due to low CDI burden in Saudi Arabia, the aim is to assess the effectiveness and outcomes of vancomycin vs metronidazole treatment options.

**Methods:**

This was a retrospective cohort study conducted in a tertiary care hospital in Jeddah which was approved by the Institutional Review Board (IRB 2020–53). Data was collected from January 2017 to April 2020. Eligible patients were adults (>18 years old) diagnosed with CDI who either received oral metronidazole (500 mg 3 times daily) or oral vancomycin (125–500 mg 4 times daily). Patients who received a combination of treatment or who were diagnosed with fulminant CDI were excluded. Demographic data were collected. The primary outcome was to assess treatment response to initial therapy with oral metronidazole versus oral vancomycin. Secondary outcomes included assessing early treatment response, time to discharge after diagnosis, proportion of patients with a positive VRE surveillance culture within 6 months of diagnosis, 30-day recurrence and 30-day all-cause mortality. Chi-square or Fisher's exact test were used to examine differences in categorical variables while student t-test or Mann–Whitney test, were used to examine differences in continuous variables. *P* value < 0.05 was considered as significant.

**Results:**

A total of 166 patients were included in the analysis. Demographic characteristics were not significantly different between the two groups. There was no difference in treatment response between vancomycin and metronidazole (96.4 % versus 94.3 %, *p* = 0.682). However, compared with metronidazole, vancomycin treatment was significantly associated with better early response (94.0 % versus 77.8 %, *p* = 0.008). Other outcomes were not significantly different between the two drug groups for time to discharge after diagnosis (*P* = 0.522), 30-day recurrence (*P* > 0.99) and 30-day all-cause mortality (*P* = 0.782). Of note, the vancomycin versus metronidazole use before the 2017 IDSA guidelines (26 % versus 74 %) was completely reversed after the release of the guidelines (83.3 % versus 16.7 %), *p* < 0.001).

**Conclusion:**

The results of this study demonstrate that vancomycin and metronidazole have comparable outcomes in regards to treatment response for non-fulminant CDI. The study also reveals the high and quick impact of international guidelines on local prescription patterns. Further studies are needed in Saudi Arabia to guide the treatment of CDI.

## 1 Introduction

*Clostridioides difficile* is a leading cause of health care-associated infections and poses an important public health threat. *C. difficile* has been associated with substantial morbidity and mortality globally and among people of all ages [1].

According to a recent meta-analysis which gathered data from 41 countries, the estimated overall incidence rate was 3.54 per 10,000 patient-days. In North America, the incidence was 6.36 per 10,000 patient-days [2]. The prevalence of *C. difficile*-associated diarrhea (CDAD) is low in Saudi Arabia in comparison to the prevalence of CDAD globally. Which was reported as 2.4 and 1.7 per 10,000 patient days in 2007 and 2008, respectively [3], while a lower prevalence rate of 0.2–0.3 per 10,000 patient days were reported from 2011 to 2012 in another center [4]. Although a more recent study done in Saudi Arabia, showed that the incidence rate is 3.5 per 10,000 patient days, it is still much lower compared to the NHSN rate of 7.2 per 10,000 patient days [5]. Moreover, the prevalence of *Clostridioides difficile* infection (CDI) in the Persian Gulf region in western Asia is lower than southern and eastern region of Asia [6]. Notably, according to a recent meta-analysis done in Saudi Arabia, the rate of vancomycin and metronidazole antibiotic resistance was very low among clinical and non-clinical isolates of *C*. *difficile* [7].

Recommendations of the 2017 IDSA guidelines regarding initial treatment of *Clostridioides difficile* [8] were based on studies conducted in the United States, Canada, Australia and Europe [[9], [10], [11]]. It is uncertain if these findings can be applicable to all patients around the globe, due to several studies that showed varying degrees of drug resistance of *Clostridioides difficile* isolates in different countries [12].

To the best of our knowledge, no studies have been conducted in Saudi Arabia to compare prescribing patterns for the treatment of *Clostridioides difficile* before and after the release of the 2017 IDSA guidelines. Considering Saudi Arabia has a low burden of CDI and the recent release of data regarding the use of metronidazole, our aim from this study was to assess the effectiveness and outcomes of vancomycin vs metronidazole monotherapy in non-fulminant CDI. This study will also provide insights with regards to antimicrobial stewardship implications and patient care in terms of the most optimum treatment for the patients in Saudi Arabia.

## 2 Materials and methods

### 2.1 Study design

This was a retrospective cohort study that was conducted in a 300-bed tertiary care hospital in Jeddah, Saudi Arabia. The study was approved by the King Faisal Specialist Hospital and Research Center Institutional Review Board (IRB 2020–53).

Data collection was performed by pharmacists and was extracted from the computerized physician order entry (CPOE) system from Jan 2017 to Apr 2020.

Eligible patients for inclusion in the study were adults (>18 years old) with an initial therapy for CDI and who received oral metronidazole (500 mg 3 times daily) or oral vancomycin (125–500 mg 4 times daily) for treatment.

A diagnosis of CDI was made by either a positive toxin enzyme immunoassay test or polymerase chain reaction test for the first episode only. Stool specimens were initially tested using the *C. diff* Quik Chek Complete® dual-antigen EIA test (D-EIA; TechLab, Blacksburg VA, U.S.A.), and the discordant results were confirmed by Xpert *C. difficile* PCR assay (Cepheid, Sunnyvale CA, U.S.A.) following the manufacturer's instructions. Specimens which provided discordant results (i.e., positive for antigen and negative for toxin) were reflex tested using the Xpert *C. difficile* PCR assay [13]. VRE screening was done by rectal swab using the GeneXpert vanA/vanB assay (Cepheid, Sunnyvale CA, U.S.A.). For the whole study period, the same diagnostic test and method was used. All patients included were symptomatic because only loose (diarrheal) stool samples are accepted for *C. difficile* testing whereas formed stool samples are rejected according to the policy in the microbiology laboratory.

Patients who received treatment of combination of metronidazole and vancomycin or those who were diagnosed with fulminant CDI were excluded.

### 2.2 Outcomes

The primary outcome was to assess the proportion of treatment response to initial therapy with metronidazole versus vancomycin in patients with non-fulminant CDI. Treatment response was defined as an improvement in CDI signs and symptoms (resolution of diarrhea and resolution of fever) by day 10 of therapy or discharge from hospital at day 10 or earlier to complete their treatment regimen as outpatient. Non-severe CDI was defined as having a white blood cell count ≤15,000 cells/mL and serum creatinine <132 μmol/L. Severe CDI was defined as having white blood cell count >15,000 cells/mL and/or serum creatinine ≥132 μmol/L. Fulminant colitis was defined as having hypotension or shock, ileus, or toxic megacolon [7].

The secondary outcomes included assessing the proportion of early treatment response (early treatment response was defined as a decrease in the number of watery stools to <3/day at 72 h), time to discharge after CDI diagnosis, proportion of patients with a 30-day CDI recurrence, proportion of patients with a 30-day all-cause mortality, and proportion of patients who had a positive VRE surveillance culture within 6 months of diagnosis.

### 2.3 Sample size

A sample of all patients was taken from Jan 2017 to Apr 2020 from the CPOE system. A list was provided by the Information Technology (IT) department in which all eligible patients were included by convenience sampling technique.

### 2.4 Statistical analysis

All categorical variables were presented as frequencies and percentage while continuous variables were presented as means and standard deviations (SD) or median and interquartile range (IQR), as appropriate.

Demographic and clinical characteristics as well as outcomes were compared between those who used vancomycin and metronidazole as the only drug treatment for CDI. Chi-square or Fisher's exact test, as appropriate, were used to examine differences in categorical variables while student t-test or Mann–Whitney test, as appropriate, were used to examine differences in continuous variables.

All *P*-values were two-tailed. *P*-value <0.05 was considered as significant. SPSS software (release 23.0, Armonk, NY: IBM Corp) was used for all statistical analyses.

## 3 Results

**Fig. 1:**
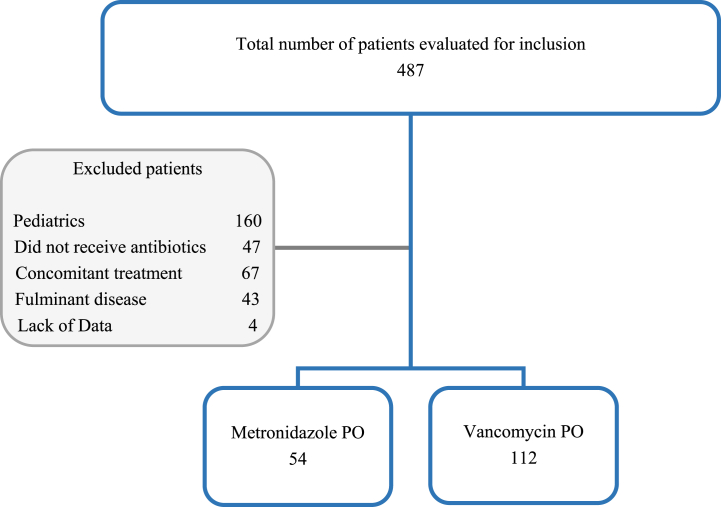
Flow diagram of case selection.

A total number of 487 patients were screened and 166 patients with an initial diagnosis of *Clostridioides difficile* infection (CDI) were included in this analysis (Fig. 1). Demographic characteristics of the patients are shown in Table 1. Approximately 67.5 % of the patients were treated with vancomycin and 32.5 % were treated with metronidazole. The mean age was 52.8 ± 17.9 years. Approximately 25.9 % were above the age of 65. Females were slightly more represented than males (54.8 % versus 45.2 %, respectively). The mean body mass index (BMI) was 25.5 ± 5.8 and approximately 23.0 % of the patients were considered obese. Compared with metronidazole, vancomycin treatment was significantly associated with younger age (50.0 ± 19.2 versus 58.7 ± 13.0, p = 0.003). Other demographic characteristics were not significantly different between the two drug groups.

**Table 1:** Demographic characteristics by type of drug treatment of CDI.

	Oral Vancomycin (N = 112)	Oral Metronidazole (N = 54)	Total (N = 166)	*P*-value
**Age in years** (mean ± SD)	50.0 ± 19.2	58.7 ± 13.0	52.8 ± 17.9	0.003
**Age groups**				
≤65	88 (78.6 %)	35 (64.8 %)	123 (74.1 %)	0.058
>65	24 (21.4 %)	19 (35.2 %)	43 (25.9 %)	
**Gender (**Female**)**	61 (54.5 %)	30 (55.6 %)	91 (54.8 %)	0.895
**Weight** (kg)	64.4 ± 15.7	67.3 ± 16.7	65.3 ± 16.0	0.277
**Height** (kg)	160.0 ± 9.6	160.3 ± 8.8	160.1 ± 9.3	0.825
**Body mass index** (BMI)	25.1 ± 5.6	26.2 ± 6.1	25.5 ± 5.8	0.258
**BMI group**				
Normal	60 (54.1 %)	20 (37.0 %)	80 (48.5 %)	0.121
Overweight	28 (25.2 %)	19 (35.2 %)	47 (28.5 %)	
Obese	23 (20.7 %)	15 (27.8 %)	38 (23.0 %)	

CDI, *Clostridioides difficile* infection; SD, standard deviation; Chi, Chi square.

Clinical characteristics of the patients are shown in Table 2. CDI was non-severe in 56.6 % of the patients and severe in 43.4 % of the patients. Baseline laboratory values included temperature of 37.2 ± 0.9 C°, while blood cell (WBC) count of 8.1 (4.7–12.3) thousands/mL, serum creatinine of 76 (46–164) umol/L, and albumin of 31.1 ± 7.5 (g/L). The average duration of therapy was 10.5 ± 4.5 days with a median (IQR) of 1 (0–2) antibiotic used during CDI course. The majority (83.7 %) of the patients had one or more comorbidity. The most frequent comorbidity was malignancy (56.1 %), followed by cardiovascular disease (44.6 %). The majority (63.9 %) of patients were using one or more antibiotics within 30 days before the CDI diagnosis. The most frequently used antibiotics were penicillins (56.6 %), followed by cephalosporins (50.9 %). Compared with metronidazole, vancomycin treatment initiation was significantly associated with a higher temperature (37.3 ± 0.9 versus 36.8 ± 0.9 C°, p = 0.001), lower WBC count (7.0 versus 8.7 thousand/mL, p = 0.040), and lower associated comorbidity (79.5 % versus 92.6 %, p = 0.032). Other clinical characteristics were not significantly different between the two drug groups.

**Table 2:** Clinical characteristics by type of drug treatment of CDI.

	Oral Vancomycin (N = 112)	Oral Metronidazole (N = 54)	Total (N = 166)	*P*-value
**CDI Severity**				
Non-severe	59 (52.7 %)	35 (64.8 %)	94 (56.6 %)	0.139
Severe	53 (47.3 %)	19 (35.2 %)	72 (43.4 %)	
**Baseline laboratory values**				
Temperature at diagnosis (Mean ± SD)	37.3 ± 0.9	36.8 ± 0.9	37.2 ± 0.9	0.001
WBC (thousands/mL) at diagnosis (Median (IQR))	7.0 (4.1–12.1)	8.7 (6.4–12.6)	8.1 (4.7–12.3)	0.040
Creatinine (umol/L) at diagnosis (Median (IQR))	76 (43–173)	75 (51–159)	76 (46–164)	0.739
Albumin (g/L) at diagnosis (Mean ± SD)	31.6 ± 7.6	30.2 ± 7.4	31.1 ± 7.5	0.262
**Number of concurrent antibiotics during CDI (**Median (IQR)**)**	1 (0–2)	1 (0–2)	1 (0–2)	0.105
**Total days of therapy (**Mean ± SD**)**	10.7 ± 4.4	10.2 ± 4.7	10.5 ± 4.5	0.505
**Comorbidity at time of diagnosis**				
No	23 (20.5 %)	4 (7.4 %)	27 (16.3 %)	0.032
Yes	89 (79.5 %)	50 (92.6 %)	139 (83.7 %)	
**Comorbidity at time of diagnosis**				
Malignancy	52 (58.4 %)	26 (52.0 %)	78 (56.1 %)	0.464
Cardiovascular disease	39 (43.8 %)	23 (46.0 %)	62 (44.6 %)	0.804
Diabetes	31 (34.8 %)	19 (38.0 %)	50 (36.0 %)	0.709
Chronic respiratory disease	7 (7.9 %)	4 (8.0 %)	11 (7.9 %)	>0.99
Liver disease	6 (6.7 %)	7 (14.0 %)	13 (9.4 %)	0.224
Dialysis	9 (10.1 %)	7 (14.0 %)	16 (11.5 %)	0.491
**Antibiotic use within 30 days before diagnosis**				
No	36 (32.1 %)	24 (44.4 %)	60 (36.1 %)	0.122
Yes	76 (67.9 %)	30 (55.6 %)	106 (63.9 %)	
**Antibiotic use within 30 days before diagnosis**				
Penicillins	42 (55.3 %)	18 (60.0 %)	60 (56.6 %)	0.658
Cephalosporins	38 (50.0 %)	16 (53.3 %)	54 (50.9 %)	0.757
Carbapenems	33 (43.4 %)	10 (33.3 %)	43 (40.6 %)	0.341
Fluoroquinolones	20 (26.3 %)	9 (30.0 %)	29 (27.4 %)	0.702
Clindamycin	5 (6.6 %)	1 (3.3 %)	6 (5.7 %)	0.673

CDI, *Clostridioides difficile* infection; SD, standard deviation; IQR, Interquartile range; WBC, white blood cells; MW, Mann-Whitney; Chi, Chi square.

Study outcomes are shown in Table 3. The median (IQR) length of hospital stay was 10 [[4], [5], [6], [7], [8], [9], [10], [11], [12], [13], [14], [15], [16], [17], [18], [19], [20], [21], [22]] days after diagnosis. The majority of the patients had treatment response (95.8 %) and early treatment response (89.0 %). Approximately 6.1 % of the patients had CDI recurrence within 30 days after diagnosis and 12.1 % died within 30 days after diagnosis. Compared with metronidazole, vancomycin treatment was significantly associated with better early response (94.0 % versus 77.8 %, p = 0.008). Other outcomes were not significantly different between the two drug groups. VRE (vancomycin-resistant Enterococci) testing performed as a rectal swab was done according to the hospital's policy for 56 patients in both groups, only 5.4 % had positive (VRE) within 6 months after diagnosis (p = >0.99), of which all positive results were in the vancomycin group.

**Table 3:** Outcomes by type of drug treatment of CDI.

	Vancomycin (N = 112)	Metronidazole (N = 54)	Total (N = 166)	*P*-value
**Treatment response**				
No	4 (3.6 %)	3 (5.6 %)	7 (4.2 %)	0.683
Yes	108 (96.4 %)	51 (94.4 %)	159 (95.8 %)	
**Early treatment response**				
No	6 (6.0 %)	10 (22.2 %)	16 (11.0 %)	0.008
Yes	94 (94.0 %)	35 (77.8 %)	129 (89.0 %)	
**Length of hospital stay after diagnosis**				
Median (IQR)	10 [[4], [5], [6], [7], [8], [9], [10], [11], [12], [13], [14], [15], [16], [17], [18], [19], [20], [21]]	8 [[3], [4], [5], [6], [7], [8], [9], [10], [11], [12], [13], [14], [15], [16], [17], [18], [19], [20], [21], [22], [23], [24], [25], [26]]	10 [[4], [5], [6], [7], [8], [9], [10], [11], [12], [13], [14], [15], [16], [17], [18], [19], [20], [21], [22]]	0.522
**Recurrence within 30 days**				
No	104 (93.7 %)	51 (94.4 %)	155 (93.9 %)	>0.99
Yes	7 (6.3 %)	3 (5.6 %)	10 (6.1 %)	
**Mortality within 30 days**				
No	97 (87.4 %)	48 (88.9 %)	145 (87.9 %)	0.782
Yes	14 (12.6 %)	6 (11.1 %)	20 (12.1 %)	

CDI, *Clostridioides difficile* infection; SD, standard deviation; IQR, Interquartile range; VRE, vancomycin-resistant *Enterococci*; MW, Mann-Whitney; Chi, Chi square.

The type of drug treatment for CDI before and after the release of the IDSA guidelines year is shown in Table 4. The vancomycin and metronidazole use before the IDSA guidelines was (10.7 % versus 63.0 %) respectively, however after the release of IDSA guidelines the use was (89.3 % versus 37.0 %, p < 0.001) in all patients.

**Table 4:** Comparison by drug treatment type for CDI before and after the release of the 2017 IDSA guidelines.

	Vancomycin (N = 112)	Metronidazole (N = 54)	Total (N = 166)	*P*-value	
Diagnosis before 2018	12 (10.7 %)	34 (63.0 %)	46 (27.7 %)	<0.001	Chi
Diagnosis between 2018 and 2020	100 (89.3 %)	20 (37.0 %)	120 (72.3 %)		
Total	112 (100.0 %)	54 (100.0 %)	166 (100.0 %)		

CDI, *Clostridioides difficile* infection; Chi, Chi square.

## 4 Discussion

CDI is recognized worldwide as a serious health threat. Although limited, there are several recent studies that showed low prevalence of CDI in Saudi Arabia [3,5,[13], [14], [15], [16]].

However, data with regard to the management of CDI in Saudi Arabia is scarce. One study that was recently conducted in a tertiary care center in the western region included data that was collected before the release of the 2017 IDSA guidelines and mentioned that metronidazole was used as first line therapy for non-severe CDI. They reported significant correlation between compliance with treatment guidelines and better clinical cure and mortality [16]. This was similar to the practice in this study in which metronidazole was used predominantly before the 2017 IDSA guidelines. It has also been observed that the use of vancomycin versus metronidazole before the release of the 2017 IDSA guidelines (26 % versus 74 %) was reversed after the release of the guidelines (83.3 % versus 16.7 %), p < 0.001). The recent changes in CDI treatment recommendations lead to an increased use of oral vancomycin. This shift in treatment recommendations for non-severe CDI was based on some studies that showed a decreased efficacy of metronidazole in comparison to vancomycin. It is presumed that this may be due to the increased incidence of CDI in the western world which resulted in an increase of its use and hence may have caused varying degrees of drug resistance [12,16,17].

On the other hand, several studies were conducted outside Saudi Arabia after the release of the 2017 IDSA guidelines. An effectiveness analysis of a cohort of US veterans less than 65 years of age with a first episode of non-severe CDI reported no difference between vancomycin and metronidazole regarding risk of 30-day all-cause mortality or CDI recurrence. However, in elderly patients or those with severe underlying comorbidities, metronidazole was inferior [18]. Another recent study showed that shifting away from metronidazole as an option in initial mild CDI did not improve the composite of treatment failure or recurrence [19].

In this study, there was no difference in treatment response between vancomycin and metronidazole (96.4 % versus 94.3 %, p = 0.682). However, compared with metronidazole, vancomycin treatment was significantly associated with better early response (94.0 % versus 77.8 %, p = 0.008). It has been reported that vancomycin reduces *C. difficile* shedding more rapidly when compared with metronidazole because it reaches higher concentrations in the stool [20]. Other outcomes were not significantly different between the two drug groups for time to discharge after diagnosis (P = 0.522), 30-day recurrence (P > 0.99) and 30-day all-cause mortality (P = 0.782). Demographic characteristics were not significantly different between the two groups. It was also noted that in patients with severe CDI, metronidazole was used in 19 patients, although hospital guidelines recommend oral vancomycin as initial treatment for such cases, further education for compliance to the guidelines will be needed as part of antimicrobial stewardship activities.

Currently, a new focused update guideline on the management of CDI in adults was released by the IDSA in June 2021 which suggested using fidaxomicin rather than vancomycin and placed vancomycin as an acceptable alternative [21]. Careful interpretation is advised before adapting new recommendations based on a different epidemiology and obtaining local data is encouraged to guide antimicrobial therapy options in Saudi Arabia. It may be prudent to continue using current antimicrobial regimens judiciously in select patients to slow down the emergence of resistance. More studies are needed locally before shifting to new antimicrobials to save these options for future use.

As of now, several guidelines still keep metronidazole as an initial treatment alternative for mild CDI [[22], [23], [24]]. Based on the current low prevalence of CDI in Saudi Arabia, it may be wise to conduct further studies in the regions and explore the option of keeping metronidazole as an alternative in treating mild CDI for low-risk patients (younger outpatients with minimal comorbidities).

Our study has several limitations including the small number of patients, which can be due to low incidence. In addition, being a retrospective study this may introduce confounders. The absence of data regarding concomitant gastrointestinal pathogens may also be considered as a limitation. This study is also single-centered which limits the generalizability to other hospitals in Saudi Arabia. Strengths of this study include the use of a 2-step diagnosis algorithm in the institution, which utilized PCR as an affirmative step for specimens with discrepancy results after the initial immunoassay test, this approach is cost-effective and provides accurate detection of toxigenic *C. difficile*. In addition, we also believe this is the first work to assess prescription patterns after the release of the 2017 IDSA guidelines in Saudi Arabia.

## 5 Conclusions

The results showed comparable outcomes between vancomycin and metronidazole regarding the treatment response of non-fulminant CDI. The study also demonstrates the high and quick impact of international guidelines which may be based on different populations on local prescription patterns. Further studies are needed in the Saudi Arabian population to guide the treatment of CDI.

## Data availability statement

The authors do not have permission to share data.

## CRediT authorship contribution statement

**Abrar F. Alhameed:** Conceptualization, Methodology, Supervision, Visualization, Writing – original draft. **Nada Saferuddin:** Data curation, Project administration, Writing – review & editing. **Tariq Alturkistani:** Data curation, Writing – review & editing. **Mohammed Al Musawa:** Writing – original draft. **Nader Damfu:** Methodology, Writing – original draft. **Majda Alattas:** Methodology, Supervision, Writing – review & editing.

## Declaration of competing interest

The authors declare that they have no known competing financial interests or personal relationships that could have appeared to influence the work reported in this paper.
